# Sensitivity of Speech Output to Delayed Auditory Feedback in Primary Progressive Aphasias

**DOI:** 10.3389/fneur.2018.00894

**Published:** 2018-10-29

**Authors:** Chris J. D. Hardy, Rebecca L. Bond, Kankamol Jaisin, Charles R. Marshall, Lucy L. Russell, Katrina Dick, Sebastian J. Crutch, Jonathan D. Rohrer, Jason D. Warren

**Affiliations:** ^1^Department of Neurodegenerative Disease, Dementia Research Centre, UCL Queen Square Institute of Neurology, University College London, London, United Kingdom; ^2^Department of Psychiatry, Faculty of Medicine, Thammasat University, Pathum Thani, Thailand

**Keywords:** delayed auditory feedback, primary progressive aphasia, semantic dementia, logopenic aphasia, Alzheimer's disease, frontotemporal dementia, dementia, progressive nonfluent aphasia

## Abstract

Delayed auditory feedback (DAF) is a classical paradigm for probing sensori-motor interactions in speech output and has been studied in various disorders associated with speech dysfluency and aphasia. However, little information is available concerning the effects of DAF on degenerating language networks in primary progressive aphasia: the paradigmatic “language-led dementias.” Here we studied two forms of speech output (reading aloud and propositional speech) under natural listening conditions (no feedback delay) and under DAF at 200 ms, in a cohort of 19 patients representing all major primary progressive aphasia syndromes vs. healthy older individuals and patients with other canonical dementia syndromes (typical Alzheimer's disease and behavioral variant frontotemporal dementia). Healthy controls and most syndromic groups showed a quantitatively or qualitatively similar profile of reduced speech output rate and increased speech error rate under DAF relative to natural auditory feedback. However, there was no group effect on propositional speech output rate under DAF in patients with nonfluent primary progressive aphasia and logopenic aphasia. Importantly, there was considerable individual variation in DAF sensitivity within syndromic groups and some patients in each group (though no healthy controls) apparently benefited from DAF, showing paradoxically increased speech output rate and/or reduced speech error rate under DAF. This work suggests that DAF may be an informative probe of pathophysiological mechanisms underpinning primary progressive aphasia: identification of “DAF responders” may open up an avenue to novel therapeutic applications.

## Introduction

Speech production is a highly complex process that depends on an interaction of motor and perceptual mechanisms. A motor programme corresponding to speech sound representations establishes perceptual predictions about the speaker's vocal output, and the motor programme is in turn updated and fine-tuned based on auditory and other perceptual feedback arising from the act of speaking (1, 2). Perturbing auditory feedback has been shown to affect vocal output. One such commonly used perturbation is delayed auditory feedback (DAF), whereby own vocal output is played back to the speaker with a slight delay (typically, between 100 and 200 ms): this leads to slowing of speech output rate and emergence of speech errors in many individuals (3–8), although there is wide variability in individual susceptibility to DAF (9, 10).

For many years, it has been recognized that DAF may paradoxically improve the fluency of speech output in stutterers, albeit with substantial individual variability (11–13). Sensitivity to DAF (i.e., reduced speech fluidity and/or accuracy in the presence of DAF) or less consistently, therapeutic benefit (improved speech fluency under DAF) have been demonstrated in various brain disorders associated with speech dysfluency, including stroke aphasia (14, 15), Parkinson's disease (16, 17), progressive supranuclear palsy (18) and autism (19). To date, however, the effects of DAF in the canonical neurodegenerative disorders of language—the primary progressive aphasias (PPA)—have not been investigated. These neurodegenerative proteinopathies are clinically, neuroanatomically and histopathologically diverse, comprising three cardinal syndromic variants: the nonfluent variant (nfvPPA), characterized by impaired speech production and agrammatism due to predominant peri-Sylvian cortical degeneration; the semantic variant (svPPA), characterized by impaired word and object knowledge due to selective anterior temporal lobe degeneration; and the logopenic variant (lvPPA), characterized by impaired phonological transcoding attributable to predominant temporo-parietal cortical degeneration (20). Although correspondences between PPA and classical vascular aphasic syndromes are loose, svPPA can be characterized as a fluent aphasic disorder, whereas nfvPPA shares several key features with Broca's aphasia. lvPPA has certain features of conduction aphasia but is also characterized by word-retrieval deficits and considerable dysfluency in a high proportion of patients (20, 21).

Certain clinical and neuroanatomical observations suggest that further exploration of the effects of DAF in PPA syndromes may be warranted. The speech slowing and speech sound errors induced by DAF in healthy individuals closely resemble the speech production deficits that characterize nfvPPA (5), hinting at a shared cognitive or pathophysiological mechanism and the possibility of therapeutic applications. Evidence in stroke populations has suggested that sensitivity to DAF may vary between aphasic syndromes, with less sensitivity in fluent and conduction aphasia albeit with considerable variability between reports (14, 22–24): this potentially opens up a novel avenue to syndrome stratification and diagnosis in the PPA spectrum, which continues to pose substantial nosological difficulties (20). Neuroanatomically, the effects of DAF and other forms of altered auditory feedback (e.g. masking, frequency shifts) on speech output are mediated by a network of areas in the healthy brain, including superior temporal, inferior parietal and prefrontal cortices (10, 25–29). Together, these regions comprise the dorsal language network which (among its principal functions) links auditory vocal representations with articulatory mechanisms and enacts sensori-motor retuning of speech production (17, 25–27, 29, 30). This dorsal network is targeted in nfvPPA and lvPPA (31), further suggesting that DAF sensitivity may be altered in these syndromes.

In this study, we assessed sensitivity to DAF in all major PPA syndromes—nfvPPA, lvPPA and svPPA—in relation to patients representing related dementia syndromes—behavioral variant frontotemporal dementia (bvFTD) and typical amnestic Alzheimer's disease (tAD)—and healthy older individuals. We scored measures of speech fluidity (output rate) and accuracy (error rate), key outcome measures that have been used in previous studies (5, 32). Since the speech of patents with nfvPPA and lvPPA is typically slow and marred by errors in the absence of DAF (20, 33), DAF effects were referenced to baseline speech output and error rates under natural auditory feedback (NAF) in all participant groups. In addition, previous work has suggested that the impact of DAF may vary depending on the context in which speaking occurs (14)—in particular, whether speech is produced spontaneously (propositional speech, as when conversing) or constrained by an external stimulus (as when reading aloud). These different kinds of speech output are neuropsychologically dissociable and may be differentially affected by brain disorders, including PPA (34–38). We therefore assessed the impact of DAF on two different speech output tasks, based, respectively on propositional speech and reading aloud.

Based on previous evidence, we hypothesized that DAF in healthy control participants would lead to slowing of speech output and appearance of speech errors during both reading aloud and propositional speech under DAF (3, 5, 14). We further predicted that PPA syndromes would show differential sensitivity to DAF, and that individual variation within groups would lead to departures from any group-wise profiles. At group level, extrapolating from previous observations in stroke aphasia (14, 22, 24), we hypothesized an overall increased sensitivity to DAF in nfvPPA, less marked sensitivity in svPPA and a mixed profile in lvPPA. We anticipated that group profiles of altered DAF sensitivity in the ‘non-aphasic' syndromes of bvFTD and tAD would be comparable to healthy older individuals. In addition, at individual level, we hypothesized that at least some patients with nfvPPA and lvPPA would show improved speech fluency under DAF (indexed by increased speech output and reduced error rates).

## Materials and methods

### Participants

Five patients with nfvPPA [three female; mean age 73.5 ± 11.4 (SD) years], eight patients with svPPA (three female; mean age 68.1 ± 7.0 years), six patients with lvPPA (one female; mean age 69.5 ± 8.5 years), 11 patients with tAD (seven female; mean age 70.0 ± 8.8 years) and eight patients with bvFTD (one female, mean age 65.6 ± 8.7 years) were recruited via a specialist cognitive clinic; 13 healthy older individuals (seven female; mean age 68.4 ± 5.4 years) with no history of neurological or psychiatric illness also participated in the study. No participant had a history of childhood stammering or clinically relevant otological disease and all had screening pure tone audiometry using a previously described procedure (39), to provide a measure of peripheral hearing function. All patients fulfilled current consensus diagnostic criteria for the relevant PPA syndrome (37), bvFTD (40) or tAD (41). Brain MRI in all patients showed an atrophy profile consistent with the clinical syndromic diagnosis and no significant concurrent burden of cerebrovascular damage. Cerebrospinal fluid tau/ beta-amyloid_1−42_ profiles were available for four of the seven patients with lvPPA, all of which were consistent with underlying Alzheimer pathology based on local reference ranges (total tau: beta-amyloid_1−42_ ratio > 1). Demographic, clinical and background neuropsychological data for all participants are summarized in Table 1.

**Table 1:** Demographic, clinical and neuropsychological characteristics of participant groups.

**Characteristic**	**Controls**	**nfvPPA**	**svPPA**	**lvPPA**	**tAD**	**bvFTD**
**DEMOGRAPHIC AND CLINICAL**						
No. (male:female)	6:7	2:3	5:3	5:1	4:7	7:1
Age (years)	68.4 (5.4)	73.5 (11.4)	68.1 (7.0)	69.5 (8.5)	70.0 (8.0)	65.6 (8.7)
Handedness (R:L)	13:0	4:1	8:0	5:1	10:1	8:0
Education (years)	17.2 (1.7)	**14.2 (2.8)**	15.1 (2.9)	15.2 (2.6)	**14.6 (1.7)**	16.0 (3.1)
MMSE (/30)	29.8 (0.4)	**25.6 (4.5)**	**24.4 (5.5)**	**19.0 (8.3)**	**17.1 (4.8)**	**17.1 (4.8)**
Symptom duration (years)	NA	3.6 (1.1)	5.6 (2.2)	3.8 (2.4)	5.5 (3.0)	6.5 (3.3)
PTA (Normal:Mild:Moderate)	3:8:0	1:2:1^a^	2:4:0^b^	2:1:2^a^	2:7:0^b^	1:5:2
**GENERAL INTELLECT**						
VIQ	127.1 (6.0)	**81.2 (18.6)**	**78.3 (14.1)**	**70.7 (16.7)**	**87.1 (14.9)**^a^	**96.8 (24.2)**
PIQ	127.0 (13.8)	**97.0 (22.3)**	121.5 (15.0)	**82.0 (12.6)**	**81.9 (18.4)**^a^	**106.3 (18.5)**
**EPISODIC MEMORY**						
RMT words (/50)	45.1 (11.0)	39.0 (6.0)^a^	**32.3 (7.4)**^a^	40.5 (7.8)^b^	**15.3 (2.8)**^a^,^*^	38.4 (10.5)^a^
RMT faces ( /50)	43.7 (4.6)	**37.0 (4.1)**^a^	**35.6 (5.4)**^a^	**36.4 (8.8)**^a^	**17.7 (2.7)**^a^,^*^	**33.1 (10.1)**^a^
**WORKING MEMORY**						
Digit span forward (max)	7.1 (1.0)	**5.4 (1.3)**	6.6 (1.4)	**4.6 (1.1)**^a^	**5.6 (1.4)**	6.7 (1.5)
Spatial span forward (max)	5.6 (0.9)^a^	5.2 (1.1)	4.9 (0.9)^a^	**3.2 (0.8)**	NA	NA
**EXECUTIVE SKILLS**						
Digit span reverse (max)	4.8 (1.3)	**2.8 (1.0)**^a^	5.0 (1.9)	**2.8 (0.8)**^a^	**3.6 (0.7)**^a^	4.4 (1.2)
Spatial span reverse (max)	5.6 (0.9)^a^	**4.2 (1.1)**	5.0 (1.0)^a^	**3.0 (1.0)**^a^	NA	NA
Letter fluency (total)	20.5 (5.5)	**6.8 (5.7)**^a^	**10.3 (4.3)**	**5.3 (5.9)**^b^	**8.4 (4.2)**	**10.5 (4.8)**
Category fluency (total)	24.8 (5.6)	**8.6 (5.9)**	21.1 (41.3)	**6.0 (9.0)**^a^	**5.3 (3.0)**	13.3 (8.5)
Trails A (s)	30.7 (8.2)	**82.8 (45.7)**	41.0 (21.9)	**106.5 (36.5)**	**95.8 (37.0)**^b^	38.3 (25.5)
**POSTERIOR CORTICAL SKILLS**						
GDA (/24)	15.8 (4.1)	**7.3 (6.6)**^b^	12.7 (7.4)^a^	**1.3 (2.3)**^c^	**1.9 (1.0)**^c^	**10.9 (7.4)**
VOSP object decision (/20)	19.2 (1.0)	17.2 (2.2)	17.5 (1.6)	**14.7 (3.1)**	**15.5 (2.3)**	17.3 (3.6)^a^
**NEUROLINGUISTIC SKILLS**						
**Speech perception**						
PALPA-3 (/36)	35.4 (0.3)	34.4 (3.0)	35.1 (0.3)^a^	**33.0 (2.2)**^a^	NA	NA
**Word retrieval**						
GNT (/30)	26.9 (2.7)	**15.8 (4.5)**	**1.0 (2.2)**	**7.4 (8.2)**^b^	**10.1 (8.4)**^a^	**15.3 (11.9)**
**Comprehension**						
BPVS (/51)	47.8 (6.3)	**35.8 (10.2)**	**13.0 (15.6)**	**26.6 (17.2)**^a^	**38.3 (5.9)**^a^	40.6 (10.0)
Concrete synonyms (/25)	24.7 (0.1)	**19.5 (4.8)**^a^	**17.3 (1.5)**^b^	**17.8 (2.4)**^a^	NA	NA
Abstract synonyms (/25)	24.6 (0.3)	**20.0 (6.0)**^a^	**16.7 (1.5)**^b^	**18.8 (1.6)**^b^	NA	NA
PALPA-55 sentences (/24)	23.9 (0.1)	**18.8 (4.9)**	**22.3 (0.9)**^a^	**16.4 (5.6)**^a^	NA	NA
**Speech repetition**						
Polysyllabic words (/45)	44.8 (0.1)	**39.8 (8.0)**	44.0 (0.6)^a^	**32.3 (11.9)**	NA	NA
Graded sentences	9.6 (0.2)	**6.0 (3.2)**	**8.3 (0.4)**^a^	**5.0 (3.8)**	NA	NA

Mean (standard deviation) values are shown; values in bold are significantly different from the healthy control group. Reduced numbers of participants are indicated:

a n-1;

b n-2;

c n-3;

* *Note that participants with tAD were given the short (25 item) RMT for both faces and words. BPVS, British Picture Vocabulary Scale; bvFTD, patient group with behavioral variant frontotemporal dementia; Controls, healthy control group; GDA, Graded Difficulty Arithmetic test; GNT, Graded Naming Test; lvPPA, patient group with logopenic variant primary progressive aphasia; nfvPPA, patient group with nonfluent-agrammatic variant primary progressive aphasia; PALPA, Psycholinguistic Assessment of Language Processing in Aphasia; PIQ, Performance IQ; PTA, pure tone audiogram (degree of hearing loss); RMT, Recognition Memory Test; svPPA, patient group with semantic variant primary progressive aphasia; tAD, patient group with typical Alzheimer's disease; VIQ, verbal IQ; VOSP, Visual Object and Space Perception battery*.

All participants gave informed consent for their involvement in the study. Ethical approval was granted by the University College London and National Hospital for Neurology and Neurosurgery Research Ethics Committees, in accordance with Declaration of Helsinki guidelines.

### Experimental procedures

**Figure 1:**
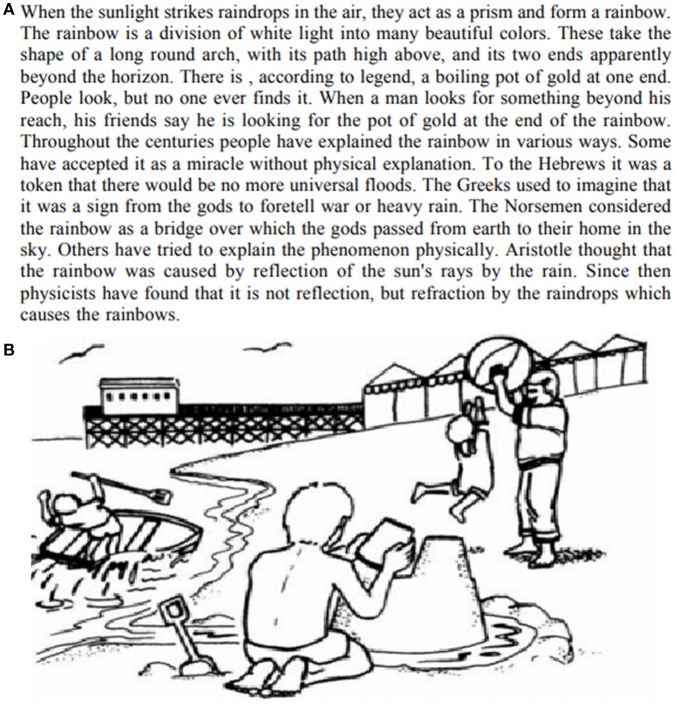
Stimuli used to elicit speech in the DAF experiment. **(A)** Reduced version of the Rainbow passage (42) used in the reading aloud task. **(B)** A beach scene [adapted from Warrington (43)] used in the propositional speech task.

All participants completed two tasks designed to assess the impact of DAF on reading aloud and on propositional speech (see Figure 1). Reading aloud was assessed using a slightly reduced version of the “Rainbow passage” (42); propositional speech was assessed by asking participants to describe a picture of a beach scene (43). For both the reading aloud and propositional speech tasks the order of NAF and DAF conditions was counterbalanced across participants, such that half of the participants in each group performed the experimental tasks first under NAF followed by DAF, while the reverse condition order was sued for the remaining half.,

The experimental paradigm was created using MATLAB v2014b® with the Psychtoolbox extension (http://psychtoolbox.org/). We adapted a script (found at http://psychtoolbox.org/docs/BasicSoundFeedbackDemo) to record sound from a boom microphone attached to the Sennheiser PC350SE® headphones worn by all participants and play this sound back (resampled at 48kHz) via the headphones. Two versions of the script were used to run the NAF and DAF conditions. In the NAF condition, the recorded sound was played back to the participant with the shortest latency supported by the 2015 MacBook Pro® computer used to run the experiment (corresponding to an imperceptible delay, typically ~18 ms, range 16–24 ms); while in the DAF condition, the recorded sound was played back with a 190 ms delay added to the minimum latency, resulting in a total delay of ~200 ms (range 190–210 ms). This latency range was chosen as corresponding approximately to the duration of a syllable in conversational spoken English and previously associated with maximal fluency disruption under DAF both in healthy individuals (3, 5, 28, 44, 45) and in patients following aphasic stroke (14). The test was administered in a quiet room, and speech samples were recorded for offline analysis.

### Scoring of speech samples

Speech samples were first edited manually in Audacity® to remove any extraneous noises during the recording and then analyzed to identify any speech sound errors, in each of five categories: (i) omissions (e.g., “rainbow” instead of “rainbows”); (ii) substitutions or misarticulations (e.g., “retraction” instead of “refraction”); (iii) duplications or additions (e.g., “sunlight-t” instead of “sunlight”); (iv) elongations (phonemes judged to have been prolonged based on the remainder of that participant's speech sample; e.g., “horiiizon” for “horizon”); (v) dysfluencies, (e.g., “um,” “er” or equivalent). In analyzing the spontaneous speech condition, we included an additional category of grammatical errors.

The total number of words produced in each condition was manually counted, and the speech output rate in words per minute (WPM) for each condition was then calculated as:

$$\begin{array}{l} \left(\text{totalnumberofwordsproduced}/\left(recordinglengthinseconds\right)\right)*60. \end{array}$$

In line with previous work (32, 5), an overall speech error rate per 100 words (PHW) was calculated as:

$$\begin{array}{l} \left(\text{totalnumberoferrorsmade}/\left(numberofwordsproduced\right)\right)*100. \end{array}$$

### Data analyses

Clinical and background neuropsychological data were analyzed using Stata v14.0®. Each patient group was compared to the healthy control group using independent-samples *t*-tests for continuous variables and chi-square tests for categorical variables.

Analyses of NAF and DAF data were run for the reading aloud and propositional speech task conditions separately. For each task condition, we derived change variables for speech output rate and speech error rate, calculated as the difference between NAF and DAF rates. Using these four change variables, each patient group was assessed for within-group change in speech output rate and speech error rate between the NAF and DAF conditions, using dependent-samples *t*-tests.

Each patient group was then compared to the healthy control group using change scores as dependent variables, in independent-sample *t*-tests directed by the result for that group from the within-group analysis. Where assumptions of the general linear model were violated, an appropriate nonparametric equivalent test was used (Wilcoxon signed-rank for dependent-samples *t*-tests; Mann-Whitney U for independent-samples *t*-tests).

All tests were two-tailed, and a statistical significance criterion thresholded at *p* < 0.05 was accepted in all cases.

## Results

### Participant group demographics and general neuropsychological characteristics

Participant groups (see Table 1) did not differ overall in terms of age [*F*_(1, 49)_ = 0.32, *p* = 0.572], handedness (χ^2^ = 5.08, *p* = 0.407), gender (χ^2^ = 7.95, *p* = 0.159), peripheral hearing ability (χ^2^ = 11.54, *p* = 0.317) or education [*F*_(1, 49)_ = 2.16, *p* = 0.148]. Patient groups did not differ in mean symptom duration [*F*_(1, 36)_ = 2.67, *p* = 0.111] or Mini-Mental State Examination score [*F*_(1, 36)_ = 0.95, *p* = 0.337; an index of overall cognitive severity].

### Effects of DAF: participant group profiles

**Figure 2:**
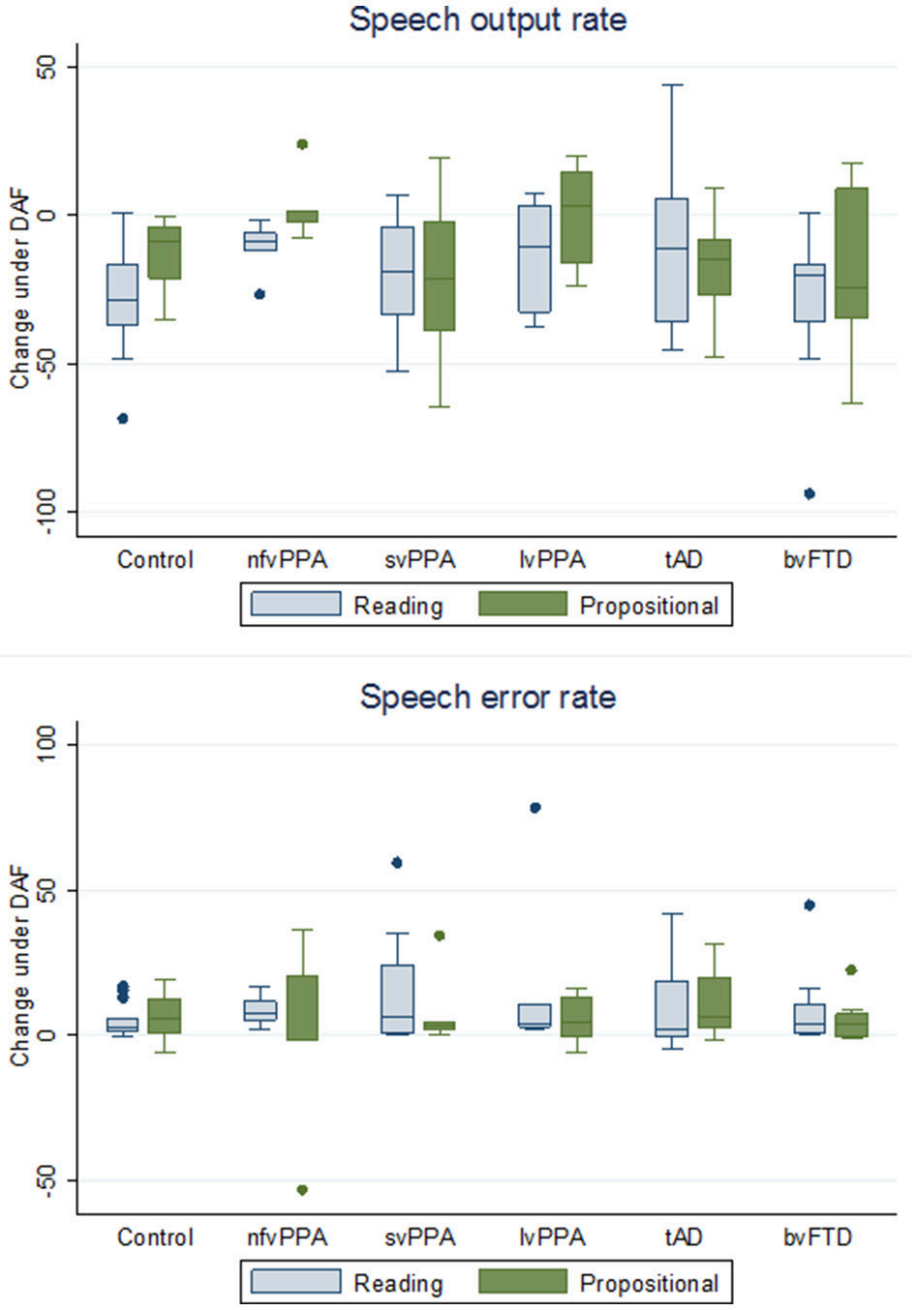
Box plots showing change in speech output rate and speech error rate under delayed audtory feedback in participant groups. The y-axis indicates changes in speech output rates and speech error rates for each participant group under delayed auditory feedback (DAF) relative to natural auditory feedback (NAF) [DAF score minus NAF score], during reading aloud and propositional speech tasks (see also Table 2): speech output rate is defined as number of words per minute; speech error rate is defined as total number of errors per 100 words. Boxes code the interquartile range and whiskers the overall range of values in each group; the horizontal line in each box represents the median. Values falling outside these ranges are indicated. bvFTD, patient group with behavioral variant frontotemporal dementia; Controls, healthy control group; lvPPA, patient group with logopenic variant primary progressive aphasia; nfvPPA, patient group with nonfluent-agrammatic variant primary progressive aphasia; svPPA, patient group with semantic variant primary progressive aphasia; tAD, patient group with typical Alzheimer's disease.

For all participant groups, speech output parameters under NAF and DAF are summarized in Table 2 and changes in speech output and speech error rates are presented in Table 3 and Figure 2.

**Table 2:** Speech output parameters under natural and delayed auditory feedback in participant groups.

**Parameter**	**Controls**		**nfvPPA**		**svPPA**		**lvPPA**		**tAD**		**bvFTD**	
	**NAF**	**DAF**	**NAF**	**DAF**	**NAF**	**DAF**	**NAF**	**DAF**	**NAF**	**DAF**	**NAF**	**DAF**
**READING ALOUD**												
Time (seconds)	79.2 (11.5)	**98.1 (23.8)**	147 (66.1)	146 (97.0)	110 (36.5)	**133 (32.5)**	156 (52.9)	183 (61.8)	107 (33.4)	114 (28.4)	87.9 (21.9)	**120 (52.3)**
Total words	216 (1.9)	217 (3.1)	160 (90.7)	130 (96.7)	201 (41.6)	200 (47.7)	223 (5.6)	220 (17.3)	213 (48.4)	208 (49.4)	215 (2.3)	213 (3.8)
Output rate	166 (23.4)	**139 (29.5)**	59.6 (18.1)	**48.6 (18.2)**	116 (35.5)	**96.2 (34.8)**	92.4 (23.2)	78.9 (23.9)	123 (33.8)	110 (27.1)	154 (36.7)	**125 (51.2)**
Error rate	1.3 (0.9)	**6.6 (5.7)**	52.7 (45.0)	**61.2 (47.3)**	5.8 (5.1)	**21.0 (24.4)**	14.9 (14.8)	**31.7 (43.9)**	15.6 (14.9)	23.2 (24.4)	2.6 (2.2)	**12.0 (16.4)**
**PROPOSITIONAL**												
Time (seconds)	45.6 (20.4)	47.2 (24.1)	59.4 (38.6)	60.8 (31.0)	68.4 (43.8)	69.3 (38.1)	76.8 (45.4)	82.0 (43.4)	62.2 (23.4)	67.3 (26.7)	51.1 (22.6)	50.9 (26.8)
Total words	105 (36.1)	100 (46.6)	43.6 (44.1)	45.6 (28.4)	129 (91.1)	113 (69.4)	102 (55.7)	114 (72.1)	118 (56.3)	107 (50.7)	105 (63.8)	95.9 (70.4)
Output rate	143 (22.2)	**129 (19.5)**	41.5 (18.9)	44.1 (8.0)	123 (42.3)	102 (26.7)	81.1 (16.5)	81.3 (14.8)	112 (23.3)	**95.8 (23.6)**	123 (52.2)	105 (43.9)
Error rate	2.9 (2.8)	**9.6 (6.2)**	38.4 (48.1)	53.9 (26.2)	3.0 (4.3)	**9.6 (15.3)**	8.6 (4.3)	14.0 (9.5)	5.2 (4.7)	**15.4 (13.5)**	3.0 (3.0)	8.4 (8.3)

*The table shows speech output rates and speech error rates in each participant group with natural auditory feedback (NAF) and delayed auditory feedback (DAF), for reading aloud and propositional speech tasks. Speech output rate is defined as number of words per minute; speech error rate is defined as total number of errors per hundred words (see Table S1 on-line for a detailed breakdown of error types). Mean (standard deviation) values are shown; values in bold indicate significant within-group differences (p < 0.05); numbers >100 are reported without decimal places to aid visual interpretation. bvFTD, patient group with behavioral variant frontotemporal dementia; Controls, healthy control group; lvPPA, patient group with logopenic variant primary progressive aphasia; nfvPPA, patient group with nonfluent-agrammatic variant primary progressive aphasia; svPPA, patient group with semantic variant primary progressive aphasia; tAD, patient group with typical Alzheimer's disease*.

**Table 3:** Change in speech fluency under delayed auditory feedback in participant groups.

	**Controls**	**nfvPPA**	**svPPA**	**lvPPA**	**tAD**	**bvFTD**
**READING ALOUD**						
Output rate	−27.6 (19.6)	−11.1 (9.5)	−19.9 (19.7)	−13.5 (18.9)	−12.3 (25.9)	−29.8 (29.1)
Error rate	5.3 (5.8)	8.6 (5.8)	15.3 (21.1)	16.8 (30.2)	7.6 (13.8)	9.4 (15.2)
**PROPOSITIONAL**						
Output rate	−13.6 (11.9)	**2.5 (12.3)**	−21.0 (27.5)	0.2 (17.6)	−16.3 (16.1)	−18.2 (28.1)
Error rate	6.6 (7.4)	−0.1 (33.8)	6.6 (11.2)	5.3 (8.5)	10.2 (10.6)	5.4 (7.7)

*The table shows changes in speech output rates and speech error rates in each participant group under delayed auditory feedback (DAF) relative to natural auditory feedback (NAF) [DAF score minus NAF score], during reading aloud and propositional speech tasks (see also Table 1 and Figure 2). Mean (standard deviation) values are shown; values in bold are significantly different from healthy controls at p < 0.05. bvFTD, patient group with behavioral variant frontotemporal dementia; Controls, healthy control group; lvPPA, patient group with logopenic variant primary progressive aphasia; nfvPPA, patient group with nonfluent-agrammatic variant primary progressive aphasia; svPPA, patient group with semantic variant primary progressive aphasia; tAD, patient group with typical Alzheimer's disease*.

#### Speech output rate

During the reading task, DAF produced significant slowing of speech output relative to NAF in healthy controls (*t* = −5.08, *p* < 0.001), in the nfvPPA group (*z* = −2.02, *p* = 0.043) and svPPA group (*t* = −2.85, *p* = 0.024) but not the lvPPA group (*z* = −1.15, *p* = 0.249); speech also slowed significantly under DAF in the bvFTD group (*z* = −2.38, *p* = 0.017) but not the tAD group (*t* = −1.57, *p* = 0.146). Slowing of speech was attributable mainly to prolongation of syllable durations rather than silent pauses. During the propositional task, DAF produced slowing of speech output in healthy controls (*t* = −3.50, *p* = 0.004) and there was a trend toward a significant effect in the svPPA group (*t* = −2.17, *p* = 0.066) but no significant effect in the nfvPPA group (*z* = −0.41, *p* = 0.686) or lvPPA group (*z* = −0.11, *p* = 0.917); speech slowed significantly under DAF in the tAD group (*t* = −3.37, *p* = 0.014) but not the bvFTD group (*t* = −3.37, *p* = 0.110).

#### Speech errors

During the reading task, DAF produced significantly more speech errors in the healthy control group (*t* = 3.29, *p* = 0.006), in all PPA syndromic groups (nfvPPA *z* = 2.02, *p* = 0.043; svPPA *z* = 2.52, *p* = 0.012; lvPPA *z* = 2.20, *p* = 0.028) and in the bvFTD group (*z* = 2.52, *p* = 0.012) but there was no significant effect in the tAD group (*z* = 1.60, *p* = 0.106). During the propositional task, DAF produced significantly more speech errors in the healthy control group (*t* = 3.14, *p* = 0.008) and svPPA group (*z* = 2.46, *p* = 0.014) but not the nfvPPA group (*z* = −0.135, *p* = 0.893) or lvPPA group (*z* = 1.36, *p* = 0.173); a DAF effect was also shown by the tAD group (*t* = 3.18, *p* = 0.010) but not the bvFTD group (*z* = 1.76, *p* = 0.079).

A detailed breakdown of error categories made by each participant group is presented in Table S1 on-line; overall, omissions were the most frequent error type induced by DAF, attributable mainly to the effect on reading aloud in the nfvPPA group. Grammatical errors during propositional speech were not significantly increased under DAF in the nfvPPA group.

### Effects of DAF: patient groups vs. healthy controls

#### Speech output rate

For the propositional task, the nfvPPA group showed significantly less slowing of speech output under DAF than healthy controls (*z* = −2.22, *p* = 0.027; see Figure 2 and Table 3); no other patient group differed significantly from healthy controls (svPPA *z* = 0.87, *p* = 0.385; lvPPA *z* = −1.49, *p* = 0.136; tAD *z* = 0.61, *p* = 0.543; bvFTD *z* = 0.36, *p* = 0.717). For the reading task, the nfvPPA group showed a trend toward significantly less slowing of speech output under DAF than healthy controls (*z* = −1.73, *p* = 0.085); no such trend relative to healthy controls was identified for any other patient group (svPPA *z* = −0.87, *p* = 0.385; lvPPA *z* = −1.14, *p* = 0.254; tAD *z* = −1.42, *p* = 0.156; bvFTD *z* = −0.22, *p* = 0.828).

#### Speech errors

Change in error rates under DAF did not differ significantly between the healthy control group and any patient group, either for reading (nfvPPA *z* = −1.23, *p* = 0.218; svPPA *z* = −0.80, *p* = 0.426; lvPPA *z* = −0.79, *p* = 0.430; tAD *z* = 0.38, *p* = 0.707; bvFTD *z* = −0.07, *p* = 0.942) or during the propositional task (nfvPPA *z* = 0.44, *p* = 0.657; svPPA *z* = 0.47, *p* = 0.657; lvPPA *z* = 0.26, *p* = 0.793; tAD *z* = −0.75, *p* = 0.451; bvFTD *z* = 0.671, *p* = 0.447).

### Individual variability in DAF response

It is noteworthy on inspection of Table 2 that the svPPA and bvFTD groups showed directional effects on speech output under DAF that were qualitatively similar to healthy controls: i.e., slowing of speech output rate and increased speech error rate during both reading and propositional speech tasks. In contrast, both the nfvPPA group and the lvPPA group showed a striking lack of effect of DAF on propositional speech rate under DAF. Individual patients within the PPA syndromic groups showed wide variation in the magnitude and direction of DAF effects on speech output and speech error rates (Figure 2); this was particularly evident for the nfvPPA and lvPPA groups, and is likely to have attenuated overall group effects relative to healthy controls.

Under DAF during the propositional task, speech output rate *increased* for two of the five individuals with nfvPPA (by 1.3 and 23.8 WPM, respectively) and three of six individuals with lvPPA (by 9.6, 19.9 and 14.8 WPM, respectively); and speech error rate *decreased* for three of five individuals with nfvPPA (−2.0, −1.7, and −53 PHW, respectively) and one of six individuals with lvPPA (−6.3 PHW). Similar “paradoxical” effects on propositional speech output were exhibited by three of eight individuals with bvFTD (by 17.3, 2.7 and 15.2 WPM, respectively) and two of 11 individuals with tAD (6.7 and 9.2 WPM, respectively), while no healthy individuals showed any such effect on propositional speech output.

## Discussion

Here we have shown that canonical PPA syndromes are associated with differential sensitivity to DAF at latency 200 ms; and that sensitivity to DAF is significantly reduced in nfvPPA, as indexed by a lack of effect on propositional speech output compared with a healthy older control cohort. Overall, both the healthy control group and most patient groups showed quantitatively or qualitatively similar profiles of DAF sensitivity, manifesting as speech slowing and increased speech error rates during propositional speech and reading aloud. The only exceptions to this pattern were exhibited by the patient groups with dysfluent PPA syndromes—nfvPPA and lvPPA—in which there was no overall slowing of propositional speech output under DAF. Our findings corroborate previous work in healthy individuals (3, 5, 10, 44, 45) but only partly substantiate studies in stroke and other aphasic syndromes (14, 22–24). Within the PPA spectrum, lvPPA shares certain features with the fluent stroke aphasia syndromes of Wernicke's and conduction aphasia, while nfvPPA most closely resembles Broca's aphasia and svPPA has no close vascular analog (20): in the stroke aphasia literature, reduced sensitivity to DAF has been documented in fluent aphasic syndromes (14, 22) whereas patients with nonfluent aphasia and speech apraxia have tended to show increased DAF sensitivity, though by no means invariably (14, 23, 24).

Based on previous behavioral and functional neuroimaging work in the healthy brain (3–8, 10, 25–27, 29), these profiles of DAF sensitivity in canonical dementia syndromes may reflect differential involvement of the dorsal language network. Dementia syndromes not primarily underpinned by damage to this network (svPPA, bvFTD, and tAD) show DAF sensitivity characteristics broadly similar to the healthy older reference group. In contrast, PPA syndromes that principally target this network might be anticipated to show altered DAF sensitivity (31, 32). While the underlying pathophysiology of DAF in these syndromes remains to be established, neurodegenerative proteinopathies and vascular insults are likely to exert fundamentally different effects on relevant neural circuits: whereas stroke interrupts neural networks focally and acutely, proteinopathies spread diffusely across networks with effects that unfold insidiously. Accordingly, syndromes of PPA and stroke aphasia might be expected *a priori* to show discrepant profiles of DAF sensitivity, reflecting the nature of the lesion they induce within the dorsal language network. The dorsal language and auditory cortical pathways behave as a functional unit, the progressive transcoding of information along these pathways supporting sensori-motor integration of speech output with auditory feedback about the speech signal produced (30, 46). If processing within these pathways is intrinsically “noisy” due to disruption of integrative computations by a neurodegenerative proteinopathy, this would make the tuning of speech output by auditory feedback less efficient and precise under both NAF and DAF and might thereby attenuate the impact of DAF relative to NAF.

In functional neuroanatomical terms, the critical mediator of DAF sensitivity may be temporo-parietal cortex, which plays an essential role in auditory-motor transformations that are fed forward to inferior frontal and other cortices governing speech output (30). The temporo-parietal junction is a core locus of pathology in lvPPA (20, 47). Whereas, involvement of inferior frontal and opercular cortices is often emphasized in formulations of nfvPPA, neuropsychological and structural and functional neuroimaging evidence has implicated additional, distributed and more posterior components of the dorsal language network and inter-connecting white matter tracts in the pathogenesis of nfvPPA (5, 20, 48–50). In line with this, recent work has highlighted more general deficiencies of complex sound analysis in nfvPPA that are not primarily motor or indeed, specifically verbal (50–55). The distributed network basis of both lvPPA and nfvPPA could potentially reconcile apparent discrepancies between our findings in PPA and previous observations concerning DAF sensitivity in aphasic stroke syndromes. Involvement of long dorsally directed white matter tracts including arcuate fasciculus would tend to interrupt auditory feedback mechanisms and reduce DAF sensitivity both in classical conduction aphasia and lvPPA (14). On the other hand, differential involvement of these pathways and their posterior connections in nfvPPA vs. Broca's aphasia would predict divergent DAF effects: more extensive involvement (in nfvPPA) would lead to blunting of DAF sensitivity, whereas sparing of feedback mechanisms (in Broca's aphasia) might amplify the more anteriorly sited primary deficit of speech production in this syndrome under DAF.

Claims to syndromic profiles of DAF sensitivity in PPA syndromes need to be heavily qualified, given the wide individual variability observed within syndromic groups. This factor (together with the relatively small cohort size) is likely to account for the absence of a significant group-level DAF effect for the lvPPA cases here. However, both the nfvPPA and lvPPA groups contained a substantial proportion of individuals who showed improvement in speech fluency under DAF: no such benefit was observed in any healthy controls. It is of interest that previous reports of therapeutic benefit from DAF have generally been based on single cases (16, 18). The mechanism of this benefit and the factors that drive variation in individual response have not been defined, though ability to compensate for DAF in healthy individuals has been shown to correlate with engagement of a distributed neural network including prefrontal, insular and parietal cortices and their subcortical connections (10): a closely overlapping network is targeted by the pathological process in nfvPPA and lvPPA (20). It is therefore plausible that amelioration of function in these damaged areas under DAF could lead to partial renormalisation of speech output in dysfluent PPA syndromes; however, this leaves open the physiological basis of the beneficial effect.

Various explanations have been proposed to account for the apparently paradoxical benefit of DAF in some people with developmental stuttering, including amelioration of an intrinsic overreliance on auditory feedback during speech output, engagement of a language mirror neuron system and regulation of speech rate (10, 13, 56). The notion that DAF regularizes the timing of articulation events by resetting and smoothing the operation of a damaged neural time-keeper might potentially account for the variable effects of DAF on speech output in developmental stuttering as well as dysfluent PPA and other neurodegenerative syndromes (such as tAD and bvFTD) in which the fine control of sensori-motor integration is vulnerable even though it is not the primary focus of pathology (56). In nfvPPA, it has been proposed that inflexible prior predictions about perceptual data generated by inferior frontal cortex lead to reduced ability to update behavior in response to sensory feedback (57): this proposal is in line with generative models of cognition that are gaining wide currency as a fundamental principle of brain operation in health as well as many disease states, and accords with the overall reduction in DAF modulation of speech output observed for the nfvPPA group here. Interestingly, Cope and colleagues (57) found (using magnetoencephalography) that patients with nfvPPA were around 200 ms slower than healthy controls in applying top-down predictions about degraded speech signals: to the extent that auditory feedback is being used to guide speech output in patents with nfvPPA, a DAF latency of 200 ms might therefore compensate for this intrinsic delay in top-down analysis of the speech signal, thereby improving speech fluency in at least some individuals with nfvPPA. On the other hand, such a mechanism would less easily account for the overall reduction in DAF sensitivity shown by the nfvPPA group here, particularly noting that patients with Broca's aphasia (and focal inferior frontal cortical damage) do seem to remain sensitive to DAF (14). As DAF always imposes a load on sensori-motor integrative mechanisms underpinning speech output, the overall impact of DAF on the disordered language system might depend on whether this load simply amplifies the effects of already “noisy” or inefficient neural processing or instead displaces an “under-damped” system into an optimized, partially compensated state. Disease-related factors (such as stage, severity, neuroanatomical and histopathological substrates) and stimulus-related factors (such as latency) could potentially influence the net effect of DAF, as could the context of speech output: constrained speech (as when reading aloud) may be particularly sensitive to DAF (13). Furthermore, both nfvPPA and lvPPA are likely to encompass separable sub-syndromes that could plausibly show distinct profiles of DAF sensitivity (20, 21, 58); any such syndromic stratification is likely to interact with consititutional factors (4) but will only be resolved by studying larger patient cohorts.

This work has several limitations which should direct future work. Case numbers in each syndromic group were relatively small; larger cohorts would increase power to demonstrate group level effects and also allow any stratification into syndromic subgroups to be more adequately assessed. Relatedly, it will be relevant to compare the effects of DAF in neurodegenerative and vascular pathologies; and to study DAF sensitivity longitudinally in patients with dementia, since it is likely a priori that syndromic profiles transform as involvement of the language system becomes more widespread and compensatory mechanisms are exhausted (in the case of genetically mediated diseases, assessing DAF sensitivity in presymptomatic individuals would be of particular interest). Measurement of objective speech parameters alone does not capture the full impact of DAF: in future work it will be important to garner patient caregiver impressions about speech fluency and quality the subjective effort associated with speaking under DAF vs. NAF (28). There are a number of other potentially relevant variables and parameters that were not assessed in our study–these include participant constitutional factors (such as a history of developmental stuttering), effects of aging, cerebrovascular and other comorbidities and stimulus factors such as DAF latency and the addition of frequency-based and other feedback manipulations. Patients with rapid, festinating speech phenotypes in the context of progressive supranuclear palsy (18) and Parkinson's disease (16) may benefit from shorter DAF latencies in the range of 50–100 ms: these phenotypes overlap clinically and pathologically with the PPA spectrum, especially nfvPPA. Moroever, different DAF latencies may access distinct pathophysiological mechanisms (10). Comparing DAF with frequency alteration and other speech feedback manipulations might illuminate underlying pathophysiological mechansims (56). Identification of underlying neural substrates will entail structural and fucntional neuroanatomical techniques; in an era of increasing interest in reversible brain dysfunction in dementia, DAF might be particularly well suited for incorporation in paradigms that address dynamic neural processes, using tools such as magnetoencephalography and transcranial magnetic stimulation (59). Ultimately, we hope that DAF will find therapeutic applications in people with PPA: to achieve this prospect, identification of the factors that charaterise “DAF responders” within syndromic groups will be crucial. Any attribution of “benefit” to DAF will need to take into account its translatability to natural communication in daily life. However, analogously with other forms of speech signal distortion 60, DAF might constitute a novel target for cholinergic and other pharmacological manipulations designed to restore sensitivity to auditory feedback in the degenerating language network.

## Conclusions

Sensitivity to DAF is reduced in nfvPPA and (less robustly) in lvPPA relative both to healthy older controls and other canonical dementia syndromes. Importantly, there was considerable individual variation in sensitivity to DAF within syndromic groups and some patients in each group (though no healthy controls) apparently benefited from DAF, showing paradoxically increased speech output rate and/or reduced speech error rate under DAF. This work identifies several areas for future clarification, notably the pathophysiological mechanisms that underpin DAF effects and the characteristics of “DAF responders” with a view to future therapeutic applications.

## Author contributions

CH, RB, KJ, and JW contributed to the conception and design of the study. CH, RB, KJ, CM, LR, KD, SC, and JR were involved in the collection and/or analysis of data. CH, RB, and JW wrote the first draft of the manuscript. All authors contributed to manuscript revision, and read and approved the submitted version.

### Conflict of interest statement

The authors declare that the research was conducted in the absence of any commercial or financial relationships that could be construed as a potential conflict of interest.
