# Supplementary material for: Sensitivity of Speech Output to Delayed Auditory Feedback in Primary Progressive Aphasias
Source: Front Neurol. 2018 Oct 29;9:894. doi: 10.3389/fneur.2018.00894 (PMC6216253; doi:10.3389/fneur.2018.00894)
Supplement: Supplementary file 1 [file Table_1.DOCX]

**Supplementary Material. Reduced sensitivity to delayed auditory feedback in nonfluent primary progressive aphasia,**

**by CJD Hardy et al**

| **Parameter** | **Control** | | **nfvPPA** | | **svPPA** | | **lvPPA** | | **tAD** | | **bvFTD** | |
| --- | --- | --- | --- | --- | --- | --- | --- | --- | --- | --- | --- | --- |
|  | NAF | DAF | NAF | DAF | NAF | DAF | NAF | DAF | NAF | DAF | NAF | DAF |
| **Reading aloud** |  |  |  |  |  |  |  |  |  |  |  |  |
| Total error rate | 1.3 (0.9) | **6.6 (5.7)** | 52.7 (45.0) | **61.2 (47.3)** | 5.8 (5.1) | **21.0 (24.4)** | 14.9 (14.8) | **31.7 (43.9)** | 15.6 (14.9) | 23.2 (24.4) | 2.6 (2.2) | **12.0 (16.4)** |
| Omissions | 0.4 (0.5) | 0.5 (0.8) | 16.7 (16.7) | **27.8 (30.7)** | 0.7 (0.9) | 3.3 (7.6) | 1.6 (1.7) | **7.1 (12.4)** | 2.9 (4.4) | **4.7 (4.8)** | 0.6 (0.5) | 2.0 (2.3) |
| Distortions | 0.4 (0.4) | **1.6 (1.4)** | 22.9 (30.4) | 23.3 (21.4) | 3.3 (4.6) | **5.4 (6.4)** | 8.7 (11.9) | 10.5 (14.7) | 5.7 (5.5) | 6.6 (5.8) | 0.7 (0.8) | 1.3 (1.4) |
| Additions | 0.4 (0.5) | **2.0 (2.6)** | **12.4 (10.0)** | 7.8 (8.5) | 1.1 (0.9) | 2.3 (2.0) | 4.3 (6.5) | **6.5 (4.5)** | 5.9 (5.4) | 10.1 (15.8) | 1.0 (0.9) | 1.2 (1.6) |
| Elongations | 0.1 (0.2) | **2.5 (3.0)** | 0.6 (0.9) | 2.5 (3.5) | 0.7 (0.8) | 10.1 (12.9) | 0.4 (0.3) | **7.7 (15.9)** | 1.2 (2.0) | 1.8 (2.7) | 0.3 (0.3) | **7.5 (12.8)** |
| Dysfluencies | 0.0 (0.0) | 0.0 (0.0) | 2.2 (3.4) | 3.1 (6.3) | 0.1 (0.2) | 0.1 (0.3) | 0.0 (0.0) | 0.0 (0.0) | 0.4 (0.6) | 0.2 (0.5) | 0.0 (0.0) | 0.0 (0.0) |
| **Propositional** |  |  |  |  |  |  |  |  |  |  |  |  |
| Total error rate | 3.0 (2.8) | **9.6 (6.2)** | 38.5 (21.5) | 38.4 (11.7) | 3.0 (4.3) | **9.6 (15.3)** | 8.6 (4.3) | 14.0 (9.5) | 5.2 (4.7) | **15.4 (13.5)** | 3.0 (3.0) | 8.4 (8.3) |
| Omissions | 0.1 (0.4) | 0.0 (0.1) | 4.9 (7.2) | 6.1 (9.3) | 0.4 (0.8) | 0.5 (1.0) | 0.5 (0.7) | 1.2 (2.8) | 0.3 (1.0) | 0.8 (2.0) | 0.1 (0.3) | 0.4 (1.0) |
| Distortions | 0.4 (0.6) | **1.3 (1.4)** | 11.3 (9.9) | 14.8 (18.8) | 0.5 (0.8) | 1.1 (0.8) | 3.8 (4.7) | 5.9 (6.5) | 1.1 (1.2) | **4.0 (3.4)** | 0.5 (0.7) | 2.5 (4.6) |
| Additions | 0.8 (1.2) | 1.9 (2.1) | 22.3 (49.0) | 15.5 (28.8) | 1.6 (3.2) | **3.0 (4.5)** | 4.3 (4.1) | 5.4 (3.5) | 3.5 (3.6) | 8.2 (10.0) | 2.1 (3.3) | 2.4 (3.1) |
| Elongations | 1.8 (1.9) | **6.3 (4.2)** | 0.0 (0.0) | 2.1 (4.6) | 0.6 (0.8) | 5.0 (10.6) | 0.0 (0.0) | 1.5 (2.4) | 0.2 (0.5) | **2.5 (3.5)** | 0.3 (0.7) | 3.2 (4.1) |
| Dysfluencies | 3.0 (2.7) | 1.9 (2.3) | 16.4 (21.4) | 8.2 (13.2) | 4.1 (3.0) | 4.1 (3.5) | 7.0 (3.3) | 6.9 (2.9) | 4.6 (2.8) | 7.0 (6.0) | 5.9 (5.5) | 5.0 (5.7) |
| Grammatical | 0.3 (0.6) | 0.8 (1.1) | 12.0 (8.1) | 7.8 (6.9) | **8.8 (1.3)** | 2.7 (2.2) | 4.7 (1.8) | 4.1 (4.1) | 1.5 (1.5) | 3.0 (3.0) | 1.7 (1.8) | 1.5 (1.6) |

**Table S1. Speech errors made under natural and delayed auditory feedback in participant groups**

The table shows rates of all speech errors and particular error types (see text for examples) made by each participant group under natural auditory feedback (NAF) and delayed auditory feedback (DAF), during reading aloud and propositional speech tasks. Speech error rates are defined as number of errors per hundred words. Values in bold indicate significant within-group differences (p < 0.05). bvFTD, patient group with behavioural variant frontotemporal dementia; Controls, healthy control group; lvPPA, patient group with logopenic variant primary progressive aphasia; nfvPPA, patient group with nonfluent-agrammatic variant primary progressive aphasia; svPPA, patient group with semantic variant primary progressive aphasia; tAD, patient group with typical Alzheimer’s disease.
